# Sustained pain relief with dihydroergotamine in migraine is potentially due to persistent binding to 5-HT1B and 5-HT1D receptors

**DOI:** 10.1186/1129-2377-14-S1-P75

**Published:** 2013-02-21

**Authors:** S Kori, J Zhang, D Kellerman, T Armer, P Goadsby

**Affiliations:** 1 MAP Pharmaceuticals, USA; 2 Dept of Neurology, UCSF, USA

## 

Several studies show that dihydroergotamine (DHE) produces sustained migraine pain relief, measured up to 48 hours, although its serum half-life is only 10-13 hours. The extended duration of action has been attributed to an active metabolite (8'-OH-DHE) with a much longer half-life than the parent compound. However, recent pharmacokinetic studies demonstrate that DHE metabolites measured in humans are too low to have substantial clinical effect and probably do not contribute to sustained efficacy. We hypothesized that the long-lasting effect of DHE is most likely due to prolonged binding to receptors, rather than serum half-life. Therefore, to investigate the mechanism of sustained migraine pain relief observed with DHE, we compared its duration of binding to serotonin receptors vs. sumatriptan.

Duration of receptor binding is expressed as the dissociation constant (koff) of the receptor-ligand complex, measured using a competitive radioligand assay. DHE and sumatriptan were tested with human 5-HT1B and 5-HT1D receptors in this study. The dissociation half-life of DHE on 5-HT1B and 5-HT1D receptors is approximately 10 times longer than that of sumatriptan (5-HT1B: 1.38 h for DHE vs. 0.17 h for sumatriptan; 5-HT1D: 1.28 h for DHE vs. 0.09 h for sumatriptan). DHE binds to 5-HT1B and 5-HT1D receptors up to 8-14 times longer than does sumatriptan. These receptors play a key role in the acute treatment of migraine and, therefore, prolonged binding to these receptors may be a mechanism for the sustained pain relief seen with DHE during the acute treatment of migraine.
